# A Manual of Modern Surgery

**Published:** 1899-09

**Authors:** 


**A Manual of Modern Surgery.** By JOHN CHALMERS DACOSTA, M.D. [Second Edition.] Pp. 911. London: Henry Kimpton. 1898.

In the new edition of this book no attempt has been made to alter the character or to change the purpose of the manual, about which we have already spoken favourably. Some new articles have been added and many of the former ones enlarged or otherwise altered to meet modern requirements. Among them may be mentioned the following: Sections have been added upon the surgery of the liver and gall-bladder, wounds inflicted by modern projectiles, and the use of the Röntgen rays. The following modern operations have been described: Resection of the Gasserian ganglion, methods of gastrostomy, use of the Murphy button, various modern operations upon the intestines, &c., &c. All of these have been well done, and, so far as was possible in the limited space, they have been fully described. The style of the author is simple and unpretentious, and he has succeeded in producing a handy volume which well meets the requirements of students reading for examination or practitioners wishing to refresh their knowledge.

